# From Obesity-Induced Low-Grade Inflammation to Lipotoxicity and Mitochondrial Dysfunction: Altered Multi-Crosstalk between Adipose Tissue and Metabolically Active Organs

**DOI:** 10.3390/antiox12061172

**Published:** 2023-05-29

**Authors:** Gina Cavaliere, Fabiano Cimmino, Giovanna Trinchese, Angela Catapano, Lidia Petrella, Margherita D’Angelo, Lucio Lucchin, Maria Pina Mollica

**Affiliations:** 1 Department of Pharmaceutical Sciences, University of Perugia, 06126 Perugia, Italy; gina.cavaliere@unipg.it; 2 Centro Servizi Metrologici e Tecnologici Avanzati (CeSMA), Complesso Universitario di Monte Sant’Angelo, 80126 Naples, Italy; fabiano.cimmino@unina.it (F.C.); angela.catapano@unina.it (A.C.); mariapia.mollica@unina.it (M.P.M.); 3 Department of Biology, University of Naples Federico II, 80126 Naples, Italy; lidia.petrella@unina.it (L.P.); margheritadangelo.dangelo@studenti.unicampania.it (M.D.); 4 Dietetics and Clinical Nutrition, Bolzano Health District, 39100 Bolzano, Italy; lucio.lucchin@nucl.it; 5 Task Force on Microbiome Studies, University of Naples Federico II, 80138 Naples, Italy

**Keywords:** adipose tissue dysfunction, hypertrophy adipocytes, metabolic diseases, neurodegenerative disorders, mitochondria, bioactive compounds

## Abstract

Obesity is a major risk factor for several metabolic diseases, including type 2 diabetes, hyperlipidemia, cardiovascular diseases, and brain disorders. Growing evidence suggests the importance of inter-organ metabolic communication for the progression of obesity and the subsequent onset of related disorders. This review provides a broad overview of the pathophysiological processes that from adipose tissue dysfunction leading to altered multi-tissue crosstalk relevant to regulating energy homeostasis and the etiology of obesity. First, a comprehensive description of the role of adipose tissue was reported. Then, attention was turned toward the unhealthy expansion of adipose tissue, low-grade inflammatory state, metabolic inflexibility, and mitochondrial dysfunction as root causes of systemic metabolic alterations. In addition, a short spot was devoted to iron deficiency in obese conditions and the role of the hepcidin–ferroportin relationship in the management of this issue. Finally, different classes of bioactive food components were described with a perspective to enhance their potential preventive and therapeutic use against obesity-related diseases.

## 1. Introduction

In the post-COVID-19 era, the maintenance of metabolic health must be a priority. This awareness stems from evidence demonstrating an increased risk of COVID-19 severity, hospitalization, admission to an intensive care unit, and mortality in patients with concomitant comorbidities (such as diabetes, hypertension, chronic kidney disease, obesity, respiratory disease, and neoplasia) [1]. In particular, systematic reviews and meta-analysis studies revealed an overall higher prevalence of hypertension (39%), diabetes (27%), obesity (27%), and 18% mortality among hospitalized patients with COVID-19 worldwide [2]. Sadly, obesity is still one of the most important global public health issues, closely linked to a higher risk for the development of a wide range of diseases including hyperlipidemia, hypertension, cardiovascular diseases, insulin resistance, type 2 diabetes, cancer, systemic immune exhaustion, and impairment of immunometabolic homeostasis [3,4]. It is thus considered a chronic disease with important health and psycho-social consequences, affecting all age groups, all populations, and countries across all income levels. The fundamental cause of obesity is attributable to an increased intake of energy-dense foods that are high in fat and sugars, and to an increase in physical inactivity; therefore, it is a consequence of a protracted positive energy balance, in which energy intake exceeds expenditure [5]. The excessive expansion of body fat is the primary hallmark of obesity [6]. Adipose tissue has a powerful capacity to adapt to nutritional stress, and its functions exceed its limited role as a mere energy reservoir. In fact, it is an endocrine organ that, in response to excessive energy intake or nutrient deficit, modulates the synthesis and secretion of adipokines. Through these signaling molecules, the adipose tissue defines inter-organ metabolic and inflammatory communication, exerting multiple impacts on the regulation of systemic energy homeostasis [7]. The importance of this crosstalk between adipose tissue and other metabolic organs explains why adipose tissue dysfunction is a determinant of obesity-associated metabolic complications that involve several peripheral districts [8]. However, the exact molecular pathophysiology of obesity and related metabolic diseases is still poorly understood. Based on compelling scientific evidence, it can be assumed that low-grade inflammation originating from adipose tissue is the major contributing factor in the development of obesity-associated alterations [9]. In particular, the key role played by the mitochondria is ever more definite in the maintenance of both metabolic and inflammatory homeostasis [10]. Evidence shows impaired mitochondria function to be involved in adipose tissue dysfunction, as they appear implicated in the insufficient lipid storage capacity of adipocytes and in adipose tissue inflammation [11]. Mitochondria play a crucial role in adenosine triphosphate (ATP) production, in both lipid synthesis and oxidation. In addition, mitochondrial function is linked to adiponectin synthesis in adipocytes, a well-known adipokine for its effects on metabolism, including an improvement in insulin sensitivity and reduction in atherosclerotic processes and systemic inflammation [12]. These data confirm that mitochondrial function downregulation results in adipose tissue dysfunction that, in turn, is a central factor for the development of obesity-related disorders, including non-alcoholic fatty liver disease (NAFLD), insulin resistance, diabetes, cardiovascular diseases, and neurodegenerative disorders [13,14,15,16]. In this review, we give an overview of the principal comorbidities related to adipose tissue dysfunction in the main metabolically active organs, focusing on the impairment of mitochondrial function as the root cause of the metabolic disorder genesis. Furthermore, we listed and described the effects of some bioactive compounds able to remediate mitochondrial dysfunction and, therefore, considered to be potential therapeutic compounds to counteract adipose tissue dysfunction and the development of related diseases.

## 2. Adipose Tissue: From Healthy to Pathologies

### 2.1. Adipose Tissues: Types and Functions

Adipose tissue is one of the body’s most important organs. It plays a pivotal role in whole-body energy status maintenance and takes part in various metabolic processes.

Indeed, it is involved in the body’s store of excess energy, and regulation of the energy balance, through the release of different hormones and metabolites [17]. It is considered the most flexible tissue in the body, due to its ability to reassess itself in size and share, changing in adipocyte size and number, in relation to the energy flux [18]. Adipose tissue is characterized by various cell types, which secrete many cytokines, chemokines, and hormones in a coordinated manner. About one-third of the cells within this tissue are adipocytes, the remainder being represented by fibroblasts, endothelial cells, macrophages, stromal cells, immune cells, and preadipocytes [19]. To date, three types of adipose tissues have been identified, namely white, brown, and beige, which are in different specific anatomic locations across the body [20]. White adipose tissue (WAT) is the predominant form of adipose tissue found in adults, and it is one of the largest organs in the body. About 10% to 20% of the total body weight in lean adults is WAT, but in individuals with obesity, the amount can increase up to 40% to 70% [21]. WAT can be classified by location; therefore, it is defined as subcutaneous (located under the skin) and visceral/omental (located intra-abdominally, adjacent to internal organs). Subcutaneous and visceral WATs are characterized by different metabolic activities, depending on their anatomical position and mitochondrial content. Indeed, studies conducted using animal models have shown a difference in mitochondria content in two major fat pads, e.g., the epididymal adipocytes, a visceral depot of WAT, represent a major content in mitochondria compared to inguinal adipocytes, a subcutaneous depot of WAT [22]. Subcutaneous WAT represents a physiological buffer for excessive energy intake during periods of reduced energy expenditure.

Furthermore, it acts as a principal energy reserve for the storage of excess lipids [23].

Visceral fat, located in the visceral compartment, is highly metabolically active and consistently releases free fatty acids (FFAs) into the portal circulation, contributing to the development of a variety of metabolic syndrome characteristics [24]. In addition to white adipose tissue, there is brown adipose tissue (BAT) and beige or “brite” adipose tissue (having mixed characteristics of both white and brown adipose cells) in humans [20]. BAT is located in distinct anatomical regions that have been well-studied and characterized in rodents [25]. Specifically, depots have been located in the scapulae (interscapular, cervical, and axillary depots) and thoracic (mediastinal depot) areas in mice and rats. In the past, it was believed to be present exclusively in hibernating animals, small mammals, and human infants. It has recently been demonstrated that adult humans have inducible BAT levels activated by cold and the sympathetic nervous system [26]. BAT accounts for between 1 and 2% of the total fat reserves in humans and is mainly located in the cervical, axillary, and paraspinal regions [25]. It is considered to be the major site of non-shivering thermogenesis in mammals and the site for the process of diet-induced thermogenesis, as a specific brown adipocyte protein, uncoupling protein-1 (UCP1), physiologically uncouples the respiratory chain to generate heat, thereby inducing increased mitochondrial fat oxidation [20]. This contributes to the clearance of plasma triglycerides, reducing ectopic lipid storage [27]. BAT has a brown color, attributable to the high iron and cytochrome content of the extensive mitochondrial network and to blood vessels within the tissue [27]. Several experimental settings with rodents have well shown that ablation of BAT [28] and genetic invalidation of the UCP1-encoding gene [29] sensitize the organism to obesity, emphasizing the negative association between BAT and obesity. Beige fat represents the third new classification of adipose tissue, and as its name indicates, brown adipocytes appear within conventional WAT depots. Beige fat has all the morphological and molecular characteristics of classical brown adipocytes present in BAT depots and, therefore, is likely to have intrinsically similar functions. However, it is physiologically distinct from BAT since it exhibits a differential expression of genes involved in metabolism and inflammation processes [30,31]. It was initially thought that beige adipocytes were the result of the transdifferentiation of white adipocytes; however, to date, its origin remains enigmatic [32]. The implementation of strategies that increase the number of beige or brown adipocytes in mouse WAT has been suggested to counteract diet-induced obesity [33]. Therefore, the cellular composition and location of adipose deposits determine their function regarding health and metabolic diseases. It is well known that WAT, by collecting excess lipids from the blood circulation, protects other tissues against the pathological accumulation of triglycerides [34]. However, this protective role is missing when lipid accumulation capacity is impaired. In this condition, an excess of lipids infiltrates the internal organs, resulting in an ectopic accumulation of lipids, which leads to metabolic problems [35], discussed below.

### 2.2. The Adipose Tissue Remodeling and the Unhealthy Hypertrophic Adipocyte

Adipose tissue expansion is related to increased adipocyte size (hypertrophy) and number (hyperplasia). These events are generally defined as adipose tissue remodeling [18]. Adipose tissue hyperplasia is considered a “recovery mechanism” to overnutrition [36], increased adipocyte number is linked to positive cellular steps, such as increased adiponectin levels, decreased basal fatty acids, and pro-inflammatory cytokine release, immune cell recruitment, and improved insulin sensitivity. The adipocyte hypertrophy, instead, is related to harmful features and has been recognized as the main cause of energy metabolic system dysfunction and obesity and its afflictions [37,38,39]. Indeed, hypertrophic adipocytes are a recognized feature of dysfunctional white adipose tissue, characterized by alterations in pathways related to hypoxia, inflammation, adipocyte differentiation [40], and secretion of important adipokines [41]. In addition, hypertrophic adipocytes exhibit significant changes in cell metabolism, characterized by elevated basal lipolysis and increased leakage of FFAs [42]. Moreover, the secretory profile of hypertrophic adipocytes evolves towards a pro-inflammatory signature, with increased levels of interleukin-6, interleukin-8, monocyte chemoattractant protein-1 (MCP-1), and tumor necrosis factor α (TNF-α), that positively correlate with adipose cell size [41]. In this context, a pivotal role is played by MCP-1, a chemokine responsible for the recruitment of macrophage cells, by amplifying the inflammatory state of adipose tissue. In turn, the pro-inflammatory cytokines released from macrophages inhibit adipocyte differentiation and increase lipolysis [43]. In hypertrophic adipocytes, endoplasmic reticulum stress (ER), caused by nutrient overload, is responsible for the inflammatory pathways (namely, JNK-AP-1 and IkbkbeNF-kB) activation, which promote phosphorylation of the serine 307 residue in insulin receptor substrate 1 (IRS-1). This interferes with IRS–insulin receptor coupling, promotes IRS degradation, and reduces downstream insulin signaling [44]. Therefore, hypertrophic adipocytes release saturated FFAs in large quantities that in turn could activate toll-like receptor 4 (TLR4) on adipocytes and macrophage plasma membranes. TLR4 induces inflammatory pathway activation, thus amplifying insulin resistance, lipolysis, and inflammation in all adipose tissue [45]. Therefore, in the case of unhealthy obesity, fat mobilization from adipocytes is impaired, and insulin is incapable of suppressing lipolysis. The reduced ability to recruit and differentiate precursor cells into mature adipocytes associated with higher lipolytic capacity results in a subsequent ectopic accumulation of excess lipids [46]. Indeed, in this condition, the unesterified fatty acids and cholesterol spill over from large adipocytes into ectopic sites that are not designed primarily for lipid storage. This mechanism is a major trigger of lipotoxicity and metabolic pathologies [47].

### 2.3. Adipocytes Dys (Function): Mitochondria Role

Although it is widely known that the mitochondria are essential organelles for maintaining metabolic homeostasis [48], so far, their role in white adipocytes has long been neglected because of their small size and low abundance [49]. Recent evidence, instead, has led to a reconsideration of their importance in adipose tissue, giving them the role of main actors in adipose tissue dysfunction and related pathologies development [50]. The mitochondria, in addition to being involved in generating energy with oxidative phosphorylation (OXPHOS) processes, regulate lipid turnover and adipogenesis and synthesize substrates for cellular metabolism (e.g., de novo fatty acid synthesis) and adipokines. It is therefore not surprising that white adipose tissue function can be disturbed by altered mitochondria [51]. Indeed, several investigations on obesity have highlighted a reduction in mitochondrial mass and function in WAT of obese ob/ob and db/db mice [52,53,54]. In addition, downregulated mitochondrial DNA was found in the WAT from obese human subjects [55,56,57], as well as a reduction in the activities of respiratory chain complexes I–IV in mitochondria isolated from subcutaneous depots [58]. Alterations in the protein expression of the electron transport chain complexes, decreased complex I activity, and enhanced reactive oxygen species (ROS) generation were also observed [59]. The reduced mitochondrial oxidative capacity in white adipocytes in obesity may result in increased activation of the inflammatory pathway, higher oxidative damage, ER stress, reduced biogenesis, and altered mitochondrial dynamics [60]. In adipocyte dysfunction processes, high mitochondrial ROS production exerts many adverse effects on the proliferation and differentiation processes of adipocytes [61]. Indeed, mitochondrial ROS, by modulating the preadipocyte number and by inhibiting adipogenesis, might influence and limit the development of adipose tissue, favoring adipocyte hypertrophy [62]. In turn, like a snake biting its own tail, it was demonstrated that the hypertrophic adipocyte presents impairment of mitochondrial function, with a further reduction in respiratory capacity and increased oxidative stress [63]. Specifically, Baldini et al. demonstrated, using in vitro studies, that hypertrophic adipocytes exhibit a reduction in the mitochondrial activity of both complex I and II and increased ROS production [63]. In addition, studies conducted on humans, where subcutaneous and visceral histology-based adipocyte size were correlated with mitochondrial function, confirmed a decreased respiratory capacity in larger adipocytes [64]. Finally, several recent investigations highlighted that mitochondrial dysfunction in adipocytes contributes to obesity-related metabolic complications [65].

## 3. Peripheral Metabolic Complications Related to Adipose Tissue Dysfunction: Focus on Metabolic Flexibility and Mitochondrial Impairment

In this section, we focused on the multi-tissue crosstalk between adipose tissue and peripheral organs, focusing on the role of mitochondrial function and metabolic flexibility in the development and progression of disorders triggered by adipose tissue dysfunction.

Adipose tissue, the liver, and muscles are key organs that govern systemic metabolic flexibility and manage the detection, uptake, transport, storage, and consumption of nutrients through the communication mediated by endocrine signals [66]. Metabolic flexibility is the ability of an organ to adapt to fluctuations in energy requirements by rapidly and efficiently switching between oxidations of different energy substrates according to their availability. This ability is essential for maintaining energy homeostasis during periods of caloric excess or restriction and during periods of low or high energy demand. A progressive reduction in this adaptive capacity has been implicated in the development of obesity-related comorbidities [67]. Indeed, metabolic inflexibility in the adipose tissue causes impaired adipokine signaling, as well as impaired non-esterified fatty acids (NEFAs) clearance from circulation, thus triggering NEFA-mediated signaling cascades in other peripheral tissues [68,69,70]. The ability of the organs to change substrates to produce energy is closely related to their mitochondrial content and function [71,72]. The mitochondria are very dynamic intracellular organelles involved in the regulation of energy metabolism, and their ability to adapt to the utilization of available substrates is the main functional component of metabolic flexibility. Indeed, many studies support the idea that the deregulation of mitochondrial function underlies the onset of metabolic inflexibility [73]. Specifically, in a high-fat diet (HFD) condition, the mitochondrial decline limits the ability of oxidative tissues to adapt fat oxidation to fat availability, leading to lipid accumulation in non-fat tissue. These lipids interfere with the insulin signaling pathway and represent one of the key aspects that could contribute to the metabolic imbalance that leads to obesity and the onset of related diseases [74].

### 3.1. Adipose Tissue—Liver Crosstalk

In an obese condition, the increase in circulating fatty acids consequent to adipose tissue dysfunction leads to inflammation, insulin resistance (IR), and injury in the liver. The highly compromised secretion/function of the adipocyte hormones, adiponectin, and leptin, which play an important role in lipid oxidation, particularly in the liver, cause ectopic fat accumulation [75]. Lipids accumulate in the cytoplasm in the form of droplets, giving rise to lipid metabolites. It was demonstrated that not the triacylglycerol itself is harmful to the cells, but the synthesis of toxic lipid intermediates, such as diacylglycerol (DAG) and ceramides (CERs) [76]. DAG is linked to altered insulin signaling and insulin resistance through the activation of hepatic protein kinase C [77], which results in reduced insulin-stimulated phosphorylation of IRS-2 and Akt2 and, thereby, the ability to activate glycogen synthesis and decrease gluconeogenesis [78]. The role of CERs in the liver has been less studied, but it is also emerging as a potentially important pathway for hepatic insulin resistance development, probably due to the inhibition of Akt/PKB (protein kinase B), a key element in the insulin signaling pathway [79]. Moreover, it has been observed that a consequence of the increased hepatic lipid pool is the development of mitochondrial dysfunction that, in turn, exacerbates the development and progression of fatty liver disease. In NAFLD, a condition comprising a spectrum of liver diseases including hepatic lipid accumulation (steatosis), liver inflammation (steatohepatitis), fibrosis, and cirrhosis, abnormal morphological changes in the mitochondria, mitochondrial proton leak, and increased oxidative stress were observed [80,81].

However, in the obese condition, it was also observed that an increased flux of fatty acids in hepatocytes leads to increased mitochondrial fatty acid import and oxidation. Indeed, individuals with hepatic steatosis had ∼5-fold higher mitochondrial respiration, and an increased mitochondrial fatty acid oxidation, considered a protective mechanism against NAFLD progression [82]. However, increased mitochondrial respiration promotes excessive hepatic oxidative stress, due to the increased production of ROS inside mitochondria, which overwhelms antioxidant systems. Once these mechanisms fail, mitochondrial functionality decreases, resulting in the development of more severe liver damage [80].

Moreover, excessive ROS can induce lipid peroxidation of phospholipids leading to the destruction of the mitochondrial membrane, resulting in impaired function of these organelles [83]. Disrupted mitochondrial function in hepatocytes leads to the activation of apoptotic and inflammatory pathways, which trigger IR and the release of chemokines and cytokines. These, in turn, elicit an increased influx of Kupffer cells and hepatic stellate cells around dying hepatocytes, which is responsible for triggering the fibrotic processes development [84].

### 3.2. Adipose Tissue—Skeletal Muscle Crosstalk

In the obese condition, the imminent consequence of elevated plasma FFAs levels on skeletal muscle is the development of insulin resistance. Indeed, several studies have shown that the increased influx of fatty acids and lipidic intermediates, such as DAG and CERs, in skeletal muscle inhibits insulin signaling by reducing glucose type 4 (GLUT4) transporters on the myocyte membrane surface [85,86,87]. Moreover, it has been demonstrated that FFAs inhibit proximal insulin-signaling steps, such as Tyr phosphorylation of insulin receptors and IRS proteins [88]. In particular, several studies provide evidence for the involvement of protein Ser/Thr kinases, which regulate IRS function, in mediating the deleterious effects of fatty acids by increasing intracellular lipid metabolites, such as fatty acyl-CoA and DAG, and by activating protein kinase C (PKC), which in turn phosphorylates and inhibits IRS signaling in the skeletal muscle both of rodents and humans [89,90]. In addition, excessive fatty acid circulation contributes to insulin resistance by activating toll-like receptors (TLRs) [91]. TLR-4 in skeletal muscle promotes the activation of JNK and IKKβ, inflammatory signaling pathways, which is associated with a marked inhibition of insulin action due to the phosphorylation of serine residues on insulin IRS-1 and inhibition of its stimulatory phosphorylation of tyrosine residues by the insulin receptor [92]. Some reports have demonstrated downregulation of mitochondrial function and a reduction in expression of genes, which encode proteins that catalyze oxidative phosphorylation in skeletal muscle, in response to high-fat feeding or lipid infusion [93,94] and, conversely, improvement in these parameters with decreased intracellular lipids [95]. In particular, increased intramyocellular lipid content was associated with the downregulation of peroxisome proliferator-activated receptor-gamma coactivator (PGC)-1α and other genes encoding protein mitochondrial respiratory complexes I, II, III, and IV [94], resulting in impaired mitochondrial biogenesis and function [96]. Furthermore, the activity of enzymes such as carnitine palmitoyltransferase-1 (CPT-1), citrate synthase, β-hydroxyacyl-CoA dehydrogenase, and others, was found to be reduced in skeletal muscle from obese and type 2 diabetic subjects [97]. Decreased mitochondrial function implies impaired fatty acid oxidation capacity, resulting in intramuscular triglyceride accumulation [98]. Evidence exists indicating increased rather than reduced fatty acid oxidation capacity in rodent models of lipid-induced insulin resistance. The increased utilization of fatty acids likely represents a homeostatic response that attempts to compensate for the elevated availability of lipids. The increased lipid availability would not lead to lipid accumulation and insulin resistance through decreased mitochondrial fatty acid oxidative capacity, but through incomplete β-oxidation, in which a large proportion of fatty acids entering the mitochondria are only partially degraded due to the depletion of several tricarboxylic acid cycle intermediates, leading to intramitochondrial accumulation of acyl-CoAs or other metabolite intermediates that, in turn, could contribute to mitochondrial failure [99,100].

### 3.3. Adipose Tissue—Cardiac Muscle Crosstalk

Obesity-related metabolic dysfunctions do not spare cardiac muscle. The heart, along with the liver and skeletal muscle, is one of the metabolically very active organs since it needs intense energy production to generate contractile force. Indeed, the expression of ATP synthase and cellular ATP production play a key role in heart function [101,102,103], and cardiac mitochondria, beyond the regulation of energy metabolism, are known to regulate other essential cardiomyocyte functions such as contractility, ROS production, and apoptosis, as well as differentiation and development [104]. In the obese condition, the high circulating fatty acids availability increases their myocardial uptake and mitochondrial oxidation and also UCP expression. The changes in the expression of UCPs, in turn, result in augmented mitochondrial uncoupling, leading to a diminished ATP production efficiency, resulting in a lower phosphocreatine/ATP ratio and altered cardiac contractility [105,106].

In addition, it was observed that when fatty acid uptake in myocytes exceeds mitochondrial oxidative capacity, there is an increase in lipid storage instead of oxidation, resulting in a lipotoxic effect associated with contractile dysfunction [107]. Studies conducted on obese animal models highlighted a reduction in state 3 respiration (maximal respiration) of mitochondria isolated both from db/db mouse hearts using pyruvate and palmitoyl carnitine as substrates and from ob/ob mice using different respiratory substrates [108,109]. Furthermore, the heart perfusions of db/db and ob/ob mouse hearts showed increased fatty acid oxidation and decreased glucose oxidation, by increased activity of peroxisome proliferator-activated receptors (PPARs), in particular of PPARα isoforms, key controllers of nuclear gene transcription, which regulate myocardial mitochondrial fatty acid oxidation [110,111]. Further studies have demonstrated that myocardial volume of oxygen (VO_2_) consumption is increased in obese ob/ob and db/db mice, also characterized by increased fatty acid oxidation and reduced cardiac efficiency observed also in the hearts of obese humans [112,113]. In addition, increasing the supply of reducing equivalents to the respiratory chain can increase ROS production, which can induce mitochondrial uncoupling by activating UCPs [114], and oxidative damage to mitochondrial proteins involved in oxidative phosphorylation, thus also affecting ATP synthesis. Finally, the reduction in ATP synthesis can lead to a shortage of heart energy and contribute to the development of contractile dysfunction [115]. In adipose tissue dysfunction, the heart initially adapts to increases in circulating fatty acid levels by increasing PPARα expression, resulting in a compensatory increase in myocardial fatty acid uptake and β-oxidation, which is believed to limit cardiac ectopic lipid accumulation [116]. However, a continuous delivery of fatty acids to the heart can cause lipid accumulation within cardiomyocytes by increasing the intracellular pool of lipid substrates involved in non-oxidative processes, including the synthesis of triacylglycerol, DAG and CERs, which can lead to different cellular dysfunctions until cell death by apoptosis [117].

There is now strong evidence that myocardial accumulation of lipids promotes the development of insulin resistance, cardiac hypertrophy, impaired cardiac function, fatty acid-induced programmed cell death, and interstitial fibrosis [118]. Despite this strong scientific evidence, it is crucial to decipher the phenomenon of the so-called obesity paradox. A growing body of evidence from epidemiological, clinical, and preclinical studies has shown that fatty acids may be beneficial in certain heart disease conditions [119]. Dhahri et al. [120] reported that a higher polyunsaturated fatty acid content may benefit by conferring lipo-protection and reducing left ventricular hypertrophy. These data emphasize the importance that future research will have to place on the prevention of excess substrates using weight control, exercise, and an adequate diet in order to safeguard the metabolic flexibility of the myocardium and, consequently, its proper function.

### 3.4. Adipose Tissue—Brain Crosstalk

Numerous studies have shown a strong association between the increased circulation of FFAs related to adipose tissue dysfunction and neurological disorders, such as Alzheimer’s disease (AD) and other dementias [121]. It is also widely known that a high-fat diet and AD are associated with blood–brain barrier (BBB) impairment, such as increased BBB permeability, disruption to tight junction proteins, thickening in the basement membrane, and altered vessel structure in animal models [122,123].

Several studies conducted on animal models provide strong evidence that elevated FFAs levels cause hypothalamic inflammation, which is considered an early step in central nervous system dysfunction and in the development of cognitive decline [124,125,126,127]. Furthermore, it was observed that FFAs are not fully catabolized in the hypothalamus but rather accumulate as long-chain acyl-CoA esters and other bioactive lipids, e.g., DAG and CER, that, in turn, active inflammatory pathways [128,129]. In fact, in vitro experiments have demonstrated a reduction in the development of the inflammatory process in hypothalamus neurons after stimulating fatty acid catabolism [130]. Recently, it was reported that HFD is responsible for cognitive deficit genesis in rodents, associated with changes in mitochondrial morphology and reduced numbers of synapses, similar to what was observed in a mouse model for AD [131]. Similarly, it was observed that an increased FFAs circulation, subsequent to dietary fat intake, might have an important role in Parkinson’s disease (PD) etiology [132]. To date, much experimental evidence has highlighted mitochondrial involvement in brain disorders and the development of neurodegenerative diseases [133,134]. Defective mitochondrial function and increased oxidative stress have been demonstrated as playing an important role in PD pathogenesis [135]. AD brains exhibit both reductions in the number of mitochondria and impaired mitochondrial function, attributable to the loss of or dysfunction in specific electron transport chain enzymes [136].

Mitochondria are abundant in neurons, especially in the synaptic region, in order to provide the high energy demands of these cells and support the local system of protein synthesis that is necessary for synaptic plasticity [137]. Therefore, their dysfunction is directly linked to compromised synaptic plasticity and subsequent neurodegenerative disease [138,139]. It would appear that excess fatty acids may impair the function of these organelles in the synaptic region. Indeed, recent studies conducted using animal models of diet-induced obesity have shown the adverse effects of HFD consumption on brain cortex bioenergetics, particularly pronounced in the synaptic region due to the markedly impaired mitochondrial function in this area [140,141].

### 3.5. Adipose Tissue and Iron Deficiency during Obesity

Iron represents the second most abundant metal on Earth and is a trace element in the human body. However, it is essential for numerous biological processes, such as transporting oxygen to tissues and controlling some functions in cell growth and differentiation, and is a cofactor in many energy metabolism processes [142]. Two-thirds of the iron in the human body is stored in hemoglobin, and the remaining is in iron-binding proteins such as ferritin and transferrin [143]. Synthesis of the heme part of hemoglobin for red blood cell production involves the use of iron in the process called erythropoiesis, in which the main source of iron is heme iron, which is recycled from aged red blood cells by macrophages [144]. Iron introduced through the diet is mainly absorbed by intestinal enterocytes in the duodenum and proximal jejunum [145].

The first evidence of a probable link between obesity and iron deficiency goes back many years, when, in the early 1960s, there was an early report published on this issue [146]. The early studies were mostly observational, where a high prevalence of iron deficiency was described in obese children, adolescents, and adults compared with their respective normal-weight subjects [147,148,149]. The real turning point came when researchers, in addition to looking at various biochemical markers related to the iron status, also began to evaluate inflammatory markers and serum hepcidin in this context [150,151]. Hepcidin is a small peptide hormone synthesized mainly in the liver and is considered a regulator of body iron homeostasis [151]. Its function in regulating plasma iron levels derives from its ability to bind ferroportin, causing its internalization and degradation [152]. Ferroportin is the only iron transporter found in human enterocytes; it transports iron across the basolateral membrane into the bloodstream, where iron is carried by transferrin and trafficked throughout the body [153]. Thus, when the hepcidin level is elevated, absorption of dietary iron from the small intestine is downregulated due to the blockade of ferroportin and consequently, serum iron levels drop [154]. In addition to enterocytes, hepatocytes and macrophages also express the receptor for ferroportin on their surface. Hepcidin, therefore, also slows the release of recycled iron from aged red blood cells mediated by macrophages [155]. This is, precisely, the functional mechanism that outlines the bridging link between obesity and iron deficiency. The non-release of iron by macrophages is a defense mechanism in response to infection and inflammation to restrict iron availability to pathogens and prevent their replication [156]. Hepcidin release from the liver is stimulated by inflammatory cytokines such as interleukin-6 [157], which, as mentioned earlier, is released from the hypertrophic adipocytes of visceral adipose tissue, triggering the state of chronic low-grade inflammation typical of obese individuals. In fact, in overweight and obese people, both serum hepcidin and interleukin-6 are significantly higher than in normal-weight people [149,158]. A recent study showed that overweight and obese women with central adiposity have high hepcidin levels, high inflammation, and low iron status that does not improve even when iron is dietarily supplemented [159]. Furthermore, it has been observed that iron is vital for mitochondrial function. In fact, both excess and deficient iron can cause mitochondrial issues [160]. On the mitochondrial outer membrane, there is a protein called MitoNEET that is responsible for regulating iron transfer within the mitochondria, thus acting as a rate-limiting step for the electron transport chain [161]. It was shown that mice with an adipose tissue-specific overexpression of MitoNEET showed a strong expansion of white adipose tissue but without influence on insulin sensitivity [162].

Finally, but not in order of importance, iron overload and an excessive increase in lipid peroxidation caused by ROS triggers a mechanism called ferroptosis, which is an iron-dependent regulated cell death [163]. This process acts at the mitochondrial level by changing their morphology, making them wrinkled with reduced or absent cristae with serious consequences on their functionality and causing cell death [164].

## 4. Bioactive Food Components as a Target to Counteract Adipose Tissue Failure and Mitochondrial Dysfunction

The evidence reported so far has emphasized the close correlation between the failure of adipose tissue, lipotoxicity, and metabolic-associated disorders, thus highlighting the pivotal role played by mitochondria in regulating this interaction. Naturally occurring bioactive compounds (NBCs) are substances contained in foods that offer benefits to human health and could represent an effective adjuvant strategy for metabolism-related disorders.

Emerging evidence shows that certain NBCs have the ability to improve adipocyte dysfunction associated with metabolic syndrome, countering obesity by promoting adipose browning [165,166] and modulating mitochondrial activity.

### 4.1. Polyphenols

Polyphenols are secondary metabolites in plants that act as a defense against ultraviolet radiation, oxidants, and pathogens [167]. Polyphenols include a large group of compounds, including phenolic acids, flavonoids, and resveratrol. The antioxidant and anti-inflammatory properties of polyphenols are well known, and their involvement in improving mitochondrial function in adipose tissue dysfunction and correlated diseases was observed [168]. Resveratrol (3, 5, 4′ trihydroxystilbene) (RSV) is a polyphenolic compound found in different fruit species, including mulberries, raspberries, pines, peanuts, blueberries, and grapes [169]. It is one of the most studied polyphenols for its diverse metabolic benefits; in fact, its antioxidant, anti-inflammatory, and anticancer properties are well known [170,171,172]. RSV has been shown to modulate mitochondrial function and dynamics, in both in vitro and in vivo experimental models for different cell types, inducing cytoprotective effects [173]. Indeed, this polyphenol would seem to upregulate the expression of superoxide dismutase (Mn-SOD) [174,175], an antioxidant enzyme that removes superoxide anions, thus preventing and restoring mitochondrial dysfunction [176]. RSV is involved in the modulation of multiple pathways, including adipogenesis, fatty acid oxidation, lipolysis, and apoptosis in adipocytes [177,178].

Experiments conducted on the animal model for diet-induced obesity have shown that RSV protects rodents against obesity and associated metabolic disease development by promoting the mitochondrial oxidative capacity of BAT, skeletal muscle, and the liver by the AMPK-SIRT1-PGC-1α pathway [177,179,180]. Specifically, RVS inhibits various phosphodiesterases and increases cytosolic cAMP levels, which triggers Epac1/CaMKKb/AMPK/SIRT1/PGC-1a pathway activation, leading to both increased mitochondrial FFA oxidation and mitochondrial biogenesis [181,182]. In mice receiving a high-fat diet, it was observed that RSV treatment elicits mitochondrial morphological changes and increased UCP-1 expression levels, predisposing mitochondria to the uncoupling of respiration. This effect largely explains the higher energy expenditure and resistance to the weight gain observed in animal models for diet-induced obesity treated with RSV [183].

In addition, RSV administration to rats fed a high-fat diet seems to increase mitochondria content/number in hepatocytes and the upregulation of hepatic UCP 2, which is involved in the control of ROS mitochondrial production, thereby preventing NAFLD development [184]. Moreover, human studies have shown that resveratrol supplementation (150 mg/kg/day for 30 days) promotes increased mitochondrial function in skeletal muscle by reducing the risk of developing type 2 diabetes [185].

Quercetin is a flavonoid found in onions, chokeberries, black currants, apples, and cherries [186]. Several experiments showed quercetin’s ability to modulate mitochondrial function and biogenesis in hepatocytes and adipocytes [187,188]. Specifically, in hepatocytes, quercetin appears to increase the expression of the gene linked to mitochondrial biogenesis by modulating enzymes and transcription factors in the inflammatory signaling cascade and by reducing ROS production. The mitochondrial biogenic effect of quercetin in adipocytes appears to be associated with stimulating mitochondrial expression of thermogenic and fatty acid oxidation genes, mtDNA replication, and AMPK activation. Furthermore, quercetin reduces HFD-induced weight gain in mice by increasing energy expenditure, due to the increased UCP-1 mRNA expression in BAT [189] and increased gene expression associated with mitochondrial oxidative phosphorylation in WAT [190]. In addition, quercetin improves skeletal muscle mitochondrial function and improves insulin sensitivity in animal models for diet-induced obesity [191].

Curcumin, a bioactive polyphenolic component derived from turmeric rhizomes, has been shown to act at the level of different cell types, regulating mitochondrial numbers and function. Indeed, its involvement in mitochondrial biogenesis was demonstrated by its action on the cAMP/PKA/AMPK signaling pathway in the skeletal muscle of rats [192]. In addition, curcumin oral administration appeared to decrease the susceptibility of liver mitochondria and microsomes to oxidative damage in an experimental model for rabbit-induced atherosclerosis with the dietary intake of saturated fat and cholesterol [193]. Curcumin stimulates UCP1 transcription via PPARα and PPARγ in BAT from male C57 BL/6 mice fed an HFD, which was considered to be the underlying cause of the increase in energy expenditure observed in this animal model [194]. In addition, curcumin treatments (1, 10, or 20 μM) increase the expression of UCP-1 and other brown adipocyte-specific markers, such as PGC-1α and PPARγ, in a dose-dependent manner in 3 T3-L1 white adipocytes [195].

### 4.2. Fatty Acids

In addition to biologically active lipids (e.g., DAG, CER), as previously mentioned, that are capable of inhibiting or activating enzymes that directly affect the insulin pathway, there are bioactive fatty acids ingested exogenously through the diet that have been shown to influence biogenesis and mitochondrial function in various cells and tissues. Among the long-chain fatty acids, α-linoleic acid, eicosapentanoic acid (EPA), and docosahexaenoic acid (DHA) are polyunsaturated (n-3) fatty acids that have been much studied as important bioactive lipids that provide health benefits, either by changing the composition of fatty acids in tissues or by inducing cell signaling pathways [196,197,198]. Omega-3 fats are found primarily in various nuts, seeds, vegetables and certain fruits, egg yolks, white and red meats, and marine organisms.

Studies using experimental animal models and humans have shown that omega-3 polyunsaturated fatty acids (PUFAs) may be helpful in reducing obesity and related metabolic diseases, improving fat oxidation and energy expenditure, and reducing fat deposits [199,200,201]. In a rat model, the intake of an ω-3 PUFA-enriched diet reduced fat accumulation in skeletal muscle and decreased metabolic/mitochondrial efficiency, attenuating oxidative stress and insulin resistance, compared to an isocaloric high-fat diet rich in saturated fatty acids [202]. It was suggested that omega-3 PUFAs may prevent or reverse deficiencies in mitochondrial function or content in skeletal muscle, increasing the expression of the transcriptional factors of mitochondrial biogenesis, such as PGC1α and nuclear respiratory factor-1, as observed in the skeletal muscle of mice fed an HFD (60% fat) with fish oil (3.4% kcal from n-3 PUFAs) for 10 weeks [203]. An increase in mitochondrial CPT-1 expression and fatty acid oxidation was also observed in the skeletal muscle of rats fed a high-fat diet supplemented with 10% *v*/*w* omega 3 PUFA for 6 weeks [204].

Moreover, in an animal model for diet-induced obesity that was fed an omega-3-enriched diet, there was also an increase in both mitochondrial function and biogenesis observed in the liver [205,206].

Evidence shows that EPA dietary intake is able to increase mitochondrial DNA content and gene expression involved in mitochondrial biogenesis (such as PGC1α) and in thermogenic processes in inguinal adipocytes of C57BL/6 mice, thereby allowing the browning of WAT and its transformation into beige adipose tissue [207]. Furthermore, it has been reported that the mixture of EPA and DHA induces a marked stimulation of BAT thermogenic activity without changes in the UCP content. These two long-chain fatty acids seem to act in synergy on BAT thermogenesis in different ways. Specifically, EPA appears to be involved in increasing the number of mitochondria, while DHA appears to be involved in both inducing BAT hyperplasia and increased mitochondrial activity [208].

A specific class of PUFAs is conjugated linoleic acid (CLA), which is a mixture of positional and geometric isomers of linoleic acid. The two most abundant are cis-9, trans-11 (c9,t11) CLA and trans-10, cis-12 (t10,c12) CLA, which have been shown to offer benefits in relation to health maintenance and disease prevention [209,210,211]. In particular, these long-chain fatty acids are able to influence mitochondria biogenesis and function in different cell types. Indeed, skeletal muscle cells treated with CLA showed a greater number and size of mitochondrial networks caused by the induction of PGC-1α pathways [212]. In addition, evidence suggests that the c9,t11CLA dietary supplement for young rats activates the cytoprotective defense Nrf2-pathway and improves mitochondrial function in the liver, counteracting a steatosis state induced by a high-fat diet [213]. The ability of CLA to modulate mitochondrial function, oxidative stress, and the inflammatory state in HFD-treated animals was also observed in skeletal muscle tissue. Indeed, CLA supplementation increases the mitochondrial oxidative capacity in skeletal muscle tissue; nevertheless, ROS formation is minimized due to the decline in mitochondrial coupling [214]. In addition, Sprague Dawley rats treated orally with CLA have shown an improvement in mitochondrial oxidative stress, respiratory enzymes, Krebs cycle enzymes, and ATP levels after administration of acrolein, a toxic substance that induces a neurotoxic effect in rat brains [215].

Short-chain fatty acids (SCFAs) are produced via the fermentation of indigestible dietary carbohydrates (predominantly resistant starch and dietary fiber) by the intestinal microbiota. SCFAs have been shown to mediate a variety of biological activities by targeting multiple organs and tissue sites and activating orphan G-protein-coupled receptors, such as GPR43 and GPR41, which are also known as free fatty acid receptors 2 and 3 (FFAR2 and FFAR3), respectively. SCFAs may play beneficial roles in appetite regulation and lipid and glucose metabolism by epigenetically regulating related genes [216,217]. Recent evidence demonstrated that exogenous intake of SCFAs can prevent weight gain in diet-induced obese mice and overweight humans [218,219], mainly by influencing mitochondrial function related to energy production, mitochondrial biogenesis, and redox balance [220,221]. It is noteworthy that our previous study demonstrated the ability of butyrate to counteract inflammation and insulin resistance in obese mice by regulating liver mitochondrial function, efficiency, and dynamics, activating the AMPK/ACC pathway, and reducing ROS generation [222,223]. In addition, the effectiveness of butyrate in modulating the inflammatory processes and oxidative stress induced by an HFD was also recently observed in the mouse brain cortex and synaptic area. Butyrate appears to act by promoting the inefficient use of mitochondrial energy substrates, i.e., generating heat instead of ATP, and by modulation of the brain-derived neurotrophic factor pathway [141].

Finally, a growing interest is turning toward N-Acylethanolamines (NAEs), a group of fatty acids derivatives that are emerging as novel therapeutic approaches to contrast obesity and insulin resistance. NAEs, including N-palmitoylethanolamine (PEA), N-oleoylethanolamine (OEA), N-arachidonoylethanolamine (AEA, anandamide), and N-docosahexaenoylethanolamine (DHEA, synaptamide), are compounds derived from fatty acids, where the carboxylic group of the fatty acid is bound to the amino group of ethanolamine through an amide bond. They are present in different types of mammalian tissues and involved in various functions in health and disease, including inflammation and energy metabolism [224]. Evidence is accumulating that NAEs and their oxidative metabolites may be aberrantly regulated or associated with disease severity in obesity, metabolic syndrome, cancer, neuroinflammation, and liver cirrhosis [224]; however, the pathophysiological mechanisms involved that underlie their mode of action are not yet fully understood. A study conducted on Sprague Dawley rats fed a hypercaloric cafeteria diet demonstrated that the effects of PEA administration on food intake and body weight gain lead to decrease weight, liver steatosis, inflammation, and dyslipidemia.

It is conceivable that the action of PEA is similar to that of OEA, which is an activator of PPARα [225]. In addition, the role of PEA in modulating hepatic metabolic inflexibility through the regulation of mitochondrial function and efficiency in an experimental animal model for diet-induced obesity has been well highlighted [226]. OEA is known to have anorexic activity, and its administration induces satiety and reduces body weight gain by activating the hedonic dopamine pathways and increasing homeostatic oxytocin and brain histamine. In addition, OEA stimulates fatty acid uptake, lipolysis, and beta-oxidation and also promotes food intake control. Therefore, OEA proposes itself as a therapeutic agent for the management and prevention of obesity and its related comorbidities [227,228].

## 5. Conclusions

**Figure 1:**
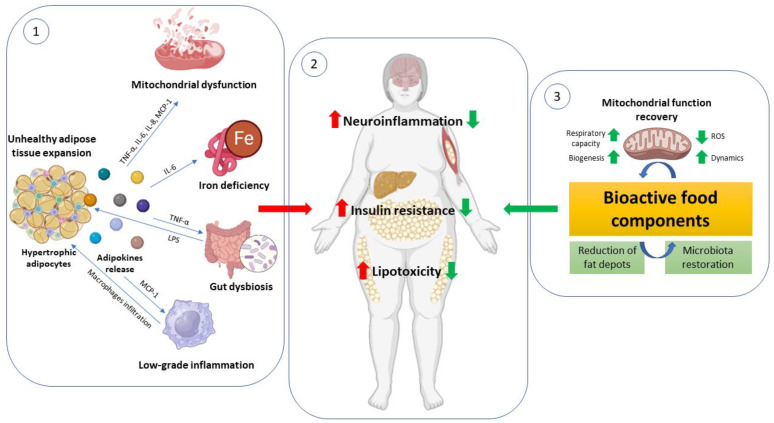
Unhealthy expansion of adipose tissue impacts mitochondrial function in metabolically active organs. ① In the obese condition, there is an unhealthy expansion of adipose tissue characterized by hypertrophic adipocytes that release numerous adipokines. Among these cytokines: MCP-1 (monocyte chemoattractant protein-1) attracts monocytes responsible for macrophage infiltration into adipose tissue and worsens chronic low-grade inflammation; TNF-α (tumor necrosis factor) with pleiotropic action increases inflammation and oxidative stress by modulating the Nf-KB pathway and mitochondrial capacity; LPS (lipopolysaccharide) derived from gut-dysbiosis contributes to systemic low-grade inflammation; IL-6 (interleukin-6) is responsible for hepcidin synthesis causing iron deficiency and ferroptosis; and finally, these adipokines impact mitochondrial function both in peripheral organs (liver, skeletal muscle, heart) and the brain. ② This context leads to increased neuroinflammation, insulin resistance, and lipotoxicity that worsen health conditions in obese subjects. ③ Some bioactive food components are able to counteract these pathological conditions through a reduction in fat depots and inflammation and an improvement in mitochondrial function with increased respiratory capacity, biogenesis, and dynamics and decreased ROS production.

In this review, we pointed out the multi-tissue crosstalk starting from the unhealthy expansion of adipose tissue as the mechanism behind the progression of obesity-related disorders such as diabetes, metabolic syndrome, cardiovascular disease, and neuroinflammation. Identifying and understanding the specific mechanisms that are altered in this scenario (mitochondrial dysfunction, iron deficiency, and intestinal dysbiosis) would provide useful targets for developing new treatments against these impairments. The bioactive food components reported in this review could be examples confirming that appropriate healthy dietary patterns represent the primary strategy to counteract metabolic alterations (Figure 1).

## Data Availability

Not applicable.
